# Prognostic and Therapeutic Potentials of OncomiRs Modulating mTOR Pathways in Virus-Associated Hepatocellular Carcinoma

**DOI:** 10.3389/fonc.2020.604540

**Published:** 2021-02-04

**Authors:** Neeti Nadda, Shashi Bala Paul, Dawesh P. Yadav, Sonu Kumar, Vishnubhatla Sreenivas, Anoop Saraya, Shivanand Gamanagatti, Subrat Kumar Acharya, Shalimar, Baibaswata Nayak

**Affiliations:** ^1^ Department of Gastroenterology, All India Institute of Medical Sciences, New Delhi, India; ^2^ Radiodiagnosis, All India Institute of Medical Sciences, New Delhi, India; ^3^ Biostatistics, All India Institute of Medical Sciences, New Delhi, India

**Keywords:** hepatocellular carcinoma, loco-regional therapy, miRNA, alpha-fetoprotein, oncomiR, hepatitis virus

## Abstract

**Background:**

Dysregulated oncomiRs are attributed to hepatocellular carcinoma (HCC) through targeting mTOR signaling pathway responsible for cell growth and proliferation. The potential of these oncomiRs as biomarker for tumor response or as target for therapy needs to be evaluated.

**AIM:**

Tumor response assessment by OncomiR changes following locoregional therapy (LRT) and targeting of these oncomiRs modulating pathway

**Methods:**

All consecutive viral-HCC patients of BCLC stage-A/B undergoing LRT were included. OncomiRs (miR-21, -221, and -16) change in circulation and AFP-ratio at 1-month post-LRT to baseline was estimated to differentiate various categories of response as per mRECIST criteria. OncomiR modulating mTOR pathway was studied by generating miR-21 and miR-221 overexpressing Huh7 stable cell lines.

**Results:**

Post-LRT tumor response was assessed in 90 viral-HCC patients (CR, 40%; PR, 31%, and PD, 29%). Significant increase of miRNA-21 and -221 expression was observed in PD (p = 0.040, 0.047) and PR patients (miR-21, p = 0.045). Fold changes of miR-21 can differentiate response in group (CR from PR+PD) at AUROC 0.718 (95% CI, 0.572–0.799) and CR from PD at AUROC 0.734 (95% CI, 0.595–0.873). Overexpression of miR-21 in hepatoma cell line had shown increased phosphorylation p70S6K, the downstream regulator of cell proliferation in mTOR pathway. Upregulation of AKT, mTOR, and RPS6KB1 genes were found significant (P < 0.005) and anti-miR-21 specifically reduced mTOR gene (P = 0.02) expression.

**Conclusions:**

The miR-21 fold change correlates well with imaging in predicting tumor response. Overexpression of miR-21 has a role in HCC through mTOR pathway activation and can be targeted.

## Introduction

OncomiRs are the microRNAs (miRNAs) targeting mostly cancer hallmark genes such as oncogene or tumor suppressor genes and extensively associated with carcinogenesis (1).These oncomiRs from tissue or circulation can be used as biomarker to predict tumor formation and outcome (2, 3). The major risk factor for hepatocellular carcinoma (HCC) is underlying chronic liver disease of viral or non-viral etiologies (4). In HCC, dysregulation of oncomiR has been implicated in tumor development and progression (5). Several miRNAs are deregulated in HCC which may have role in carcinogenesis that includes upregulation of miR-21, -122, -125a/b, -199a/b, -221, -222, -223, -224, and-500; or downregulation of miR-124 and -199 (6). OncomiRs specific to HCC are considered as promising biomarker not only for diagnosis but also for prognosis and monitoring therapeutic response. Few studies have reported prediction of tumor response following intervention in HCC using serum microRNA such as miR-181a-5p, miR-10b-3p, and miR-199-a/b-3p (7, 8). The miRNAs targeting mTOR pathway may have great potential as biomarker for tumor response prediction. Deregulation of various components of mTOR pathway has been reported in breast, ovarian, renal, colon, head, and neck cancers that includes amplification or mutation of PI3K, PTEN suppression, and overexpression of AKT, S6K1, 4EBP1, and eIF4E (9). In case of HCC, about 40–50% of tumors show dysregulated expression of several upstream and downstream effectors of mTOR, including EGF, IGFBP3, IGF2, raptor, PTEN, RPS6, and p70S6 kinase and mTOR pathway activation is found to be associated with less differentiated tumors, bad prognosis, and earlier recurrence independent of the underlying etiology of liver cancer (10).

Locoregional therapy (LRT) is the main-stay of treatment for unresectable HCC patients (11). LRT selectively destroys the tumor tissue without affecting the adjoining hepatic parenchyma and results in prolonging survival. Accurate post-LRT tumor response assessment is essential as retreatment may be required in patients with residual disease or in the case of recurrence (revascularization) of initially treated tumor. Evaluation of therapeutic response is recommended by multiphasic contrast-enhanced computed tomography (MPCT) or magnetic resonance imaging (MRI) of the liver as per the mRECIST (modified Response Evaluation Criteria in Solid Tumors) criteria (12). This response evaluation criteria categorizes tumor response into complete response (CR), partial response (PR), stable disease (SD), and progressive disease (PD) (13). Recent studies have shown the new role of serum alpha-fetoprotein (AFP) as prognostic biomarker of HCC (14–17). It is frequently measured in clinical practice during the course of HCC treatment based on the hypothesis that AFP is continuously reflective of tumor activity and burden. AFP serum level changes in predicting response to various types of LRTs in AFP secreting tumors have been documented (14, 15) but AFP is known to be secreted only in 60–80% of the HCC cases (16, 17). In consideration of above facts, this prospective study was undertaken on viral-HCC patients for evaluating changes in oncomiRs following LRT. Tumor response on imaging was also correlated with oncomiRs and serial changes in serum AFP. The specificity and functional evaluation of oncomiR was carried out with respect to several genes modulating mTOR pathway.

## Materials and Methods

### Patients and Clinical Investigations

This prospective observational study was carried out in a tertiary care setting at the Department of Gastroenterology, All India Institute of Medical Sciences, New Delhi, India during February, 2015 to August, 2018 after obtaining Institute ethics committee (IEC) approval (IESC/T121/25.02.2015). All consecutive HCC patients of viral etiology (n = 158) were included for locoregional therapy with curative intent (RFA) and palliative intent (TACE). HCC of all included patients were diagnosed as per European Association for the Study of the Liver (EASL) criteria (12, 18) and were at stage A or B as per BCLC staging classification (19, 20). All the patients have undergone RFA, TACE, and PAI as locoregional therapy. The demographic profile, clinical, radiological (abdominal ultrasound and MPCT/MRI of the liver), and biochemical parameters of all patients were recorded. Laboratory investigations for biochemical parameters were complete blood count, liver and kidney function tests, serological viral (HBV and HCV) markers, serum AFP, and serum microRNA.

### Loco-regional Therapy (LRT) and Monitoring Therapeutic Response in HCC Patients

After baseline evaluation, patients were evaluated for treatment plan on the basis of BCLC staging and appropriate LRT of either RFA, PAI, or TACE was undertaken (21). All patients were followed up at 1 month following the procedure of LRT. They were subjected to a repeat clinical, biochemical evaluation (including oncomiRs and serum AFP) to note the changes from baseline to 1 month post treatment. AFP secreting HCC patients were defined as the ones who had AFP >20 ng/ml at baseline. The AFP ratio was calculated from the AFP levels after 1 month as post/pre-treatment.

MPCT/MRI of liver was done for tumor response evaluation according to the mRECIST criteria which categorized into complete response (CR), partial response (PR), progressive disease (PD), and stable disease (SD). In our study, HCC patients were classified into two groups for comparison according to therapeutic response. This includes Group1: CR+PR *vs.* PD and Group 2: CR *vs.* PR+PD.

## miRNA Isolation and cDNA Synthesis

The miRNAs were isolated from 200 μl of serum sample using Exiqon’s miRCURY™ RNA Isolation Kit (Cat no. 300112) and from hepatic cell lines using *mir*Vana™ Kit (Cat no. AM 1560) as per manufactures protocol. The cDNA was synthesized using 25 ng of miRNA in universal cDNA synthesis kit II, 8-64 rxns (Exiqon, cat no. 203301). Briefly, mature miRNAs and synthetic spike-in were polyadenylated by poly(A) polymerase and converted into cDNA by reverse transcriptase with oligo-dT priming in same tube. The oligo dT primers were having a 3’ degenerate anchor and a universal tag sequence on the 5’ end which allowed amplification of mature miRNA in the real-time PCR step using oligo-dT primer or universal reverse primer included in the kit. The cDNA was diluted (1:40) with nuclease free water for real time PCR reaction.

### Real-Time PCR and Relative Expression of miRNA

Six miRNAs (miR-21,-221, -16, -199, -124, and -500) were selected as candidate miRNA for monitoring therapeutic response as evidenced from literature for its deregulation in HCC (3). Real-time PCR of miR-21-5p, miR-221-3p, miR-16-5p, miR-199a-3p, miR-124-3p, and miR-500a-3p was performed using ExiLENT SYBR^®^ green master mix (Cat no. 203403) and LNA (locked nucleic acid) primer (Exiqon). Briefly, the miRNA cDNA was diluted to 1:40 time and the reaction was set in 10 μl PCR volume (1× PCR Master mix, 1 μl PCR primer mix, and 4 μl of diluted cDNA) in 96-well optical plate in duplicates. The reaction condition was incubation at 95°C for 10 min, followed by 40 cycles of 95°C for 10 s; 60°C for 1 min followed by melt curve analysis. The miRNA expression in serum was expressed relative to exogenous control (Unisp6) by comparative ΔCt method expressed as 10^5^ log 2^-ΔCt^ values (22). Difference if any between miRNA expression in HBV-HCC and HCV-HCC was noted. Relative fold changes of miRNA expression at one month post- vs. pre-therapy of viral-HCC patients was calculated by comparative 2^-ΔΔCt^ method (22).

### Bioinformatic Analysis, PTEN 3′-UTR Cloning in psiCHECK-2 Reporter Vector and Luciferase Assay

The putative binding site of miR-21 and -221 targeting mTOR pathway was determined through bioinformatic analysis using miRTarbase. The PTEN gene, a negative regulator of mTOR pathway, was found to be a target and binding sites were mapped to 3’ UTR region of 177-206nt for miR-221 and 420-437nt for miR-21. The region flanking miR-221 binding sites in the PTEN 3’-UTR (152-923nt) were PCR amplified using primers 152F and 923R (**Table 1**) which contain XhoI and NotI for directional cloning. The 3′-UTR region target (420-437nt) specific to miR-21 further amplified using primers RE flanking 268F and 517R primers and cloned in the psiCHECK-2 luciferase reporter vector at XhoI/NotI site (Promega, Madison, WI, USA). Both UTR reporter vector and miRNAs (LNA based RNA Oligo) were transiently transfected to Huh7 cells using lipofectamine 2000 (Invitrogen). The cells were harvested at 48 hr post-transfection. Reporter renilla luciferase activity in transfected cells were measured in Glomax luminometer using dual-Luciferase Reporter Assay System (Promega, USA) and normalized by absorbance of viable cell determined in MTT assay.

**Table 1:** Sequence of primers used for PTEN 3’UTR cloning and Sh-miR precursors cloning.

PTEN152F	5’-aactcgagatacatccacagggttttgacact-3’
PTEN923R	5’-aagcggccgcacaggagatggagaagtcgttaca-3’
PTEN268F	5′-aactcgaggatggcactttcccgttttattcc-3′
PTEN517R	5′-aagcggccgcagcattccctccattcccctaacc 3′
**miR-21**	**miR-21-5p precursor oligo**
Oligo1	ACCTCTAGCTTATCAGACTGATGTTGATCAAGAGTCAACATCAGTCTGATAAGCTATT
Oligo2	CAAAAATAGCTTATCAGACTGATGTTGACTCTTGATCAACATCAGTCTGATAAGCTAG
**miR-221**	**miR-221-3p precursor oligo**
Oligo1	ACCTCAGCTACATTGTCTGCTGGGTTTCTCAAGAGGAAACCCAGCAGACAATGTAGCTTT
Oligo2	CAAAAAAGCTACATTGTCTGCTGGGTTTCCTCTTGAGAAACCCAGCAGACAATGTAGCTG
**miR-Scr**	**miR-Scr precursor oligo**
Oligo1	ACCTCGTGAGTCGTCAGTCAATTACTATCAAGAGTAGTAATTGACTGACGACTCACTT
Oligo2	CAAAAAGTGAGTCGTCAGTCAATTACTACTCTTGATAGTAATTGACTGACGACTCACG
PTEN Forward	5′-CCACAAACAGAACAAGATGCT-3′
PTEN Reverse	5′-GCTCTATACTGCAAATGCTATCG-3′
AKT1 Forward	5′-CTCCCCTCAACAACTTCTCTG-3′
AKT1 Reverse	5′-GCGTTCGATGACAGTGGT-3′
mTOR Forward	5′CGTGGAATTTGAGGTGAAGC 3′
mTOR Reverse	5’-AGAAGGTAGGGACGCTGAT-3′
RPS6KB1 Forward	5′-CAGTTAAATGAAAGCATGGACCA-3′
RPS6KB1 Reverse	5′- CAAGTACCCGAAGTAGCTCAA -3′
β-Actin Forward	5’GCACCACACCTTCTACAATGA 3’
β-Actin Reverse	5’ TGTCACGCACGATTTCCC 3’

### Short Hairpin microRNA (Sh-miR) Cloning in psiRNA-h7SKGFPzeo Vector for Stable miR-21, -221 and Scramble (miR-Scr) Expression

Synthetic oligos were custom designed as Sh-miR precursors using siRNA Wizard software (Invivogen, USA). Both sense and antisense DNA oligos (50nt long) for short hairpin based mature miRNA were synthesized and annealed to have BbsI overhangs for cloning in psiRNAGFPzeo vector at BbsI site. Screening for positive clones was carried out by restriction digestion using SpeI and sequence confirmed. The sequence of oligos was mentioned in **Table 1**. For stable expression in Huh7 cells, miR-21, miR-221, and miR-Scr expressing psiRNAGFPzeo plasmids were transfected using lipofectamine 2000. Antibiotic Zeocin selection for stable miR and GFP reporter expression in Huh7 cell was carried out at 500 μg/ml concentration and maintenance with 300 μg/ml of Zeocin. Overexpression of miR-21 and miR-221 from stable cells were checked by real-time PCR using miRNA specific LNA primers (exiqon).

### Functional Effect of microRNA Stable Expression in Huh7 Cell

Effect of miR-21, miR-221, and miR-Scr over expression were determined by MTT assay for cell proliferation. Briefly, cells were incubated with 5 mg/ml of MTT at 37°C for 4 h in humidified atmosphere. Formazan crystals were dissolved in DMSO and the optical density was recorded at 570 nm using ELISA reader (Multiscan Go, Thermo Fisher). Effect of miR on mTOR pathway signaling was determined by western blotting. Modulation of mTOR pathways were studied by immunoblotting using anti human- PTEN, AKT, mTOR, total and phospho (T389) p70 S6 kinase, and GAPDH. The proteins were detected with enhanced chemiluminescence reagent (Biorad).

### Relative Expression of miRNA Target Gene by Real-Time PCR and Effect of antimiR in miR Overexpressing Stable Cell Line

Total RNA was isolated from cultured mammalian cells and the cDNA was synthesized using High-capacity cDNA Reverse transcription kit (Applied biosystem). PTEN, AKT, mTOR, RPS6KB1, and beta-actin mRNA relative expression in miR-21 and miR-221 overexpressing Huh7 cell lines with or without anti-miR (50nm) treatment was studied by SYBR Green (Agilent technologies) based Real time PCR.

### Statistical Analysis

The difference between mean values of pre and post test of miR-21 was assumed to have effect size of 0.5 and a difference in standard deviation of differences 1.0 between pre and post. Therefore the required sample size for 80% power and 5% level of significance recorded sample size is 31. Considering the three possible subgroups, CR, PR, and PD, it is decided to take 30 patients in each group and total 90 patients were recruited for study. Normally distributed continuous variables were expressed as mean ± standard deviation (SD) and categorical variables were expressed as percentages. The statistical significance of relative gene expression between HBV and HCV group, pre and post therapy; categorized response groups were analyzed by t tests. Continuous variables were compared by student t-test or ANOVA test and categorical variables were compared by chi-square test and p value of <0.05 was considered to be significant. Similarly, the comparison between groups for skewed data was done by Kruskal-Wallis test, followed by Mann Whitney test with adjusted p-values. The trends in changes of oncomiR following treatment were correlated with the tumor response of CR, PR, and PD assessed on MPCT/MRI at 1 month post-LRT. Changes in AFP ratio of AFP secreting (>20 ng/ml) patients were estimated in different categories of tumor response. Two groups (Group 1 and 2) were categorized according to the therapeutic response. Group 1 was categorized to differentiate CR+PR from PD patients and Group 2 was categorized to differentiate CR from PR +PD patients by drawing ROC curves on the basis of relative fold changes of miRNA and AFP ratio at one-month post-LRT. Overall survival was defined as the time interval from the date of LRT to death or censored on the last 42 month post-LRT follow-up. Survival proportions and Kaplan-Meier curve comparisons among groups were calculated using log-rank test (Mantel-Cox test) and considered significant if *p* **< **0.05. The luciferase activity and gene expressions between two groups were compared by unpaired t test.

## Results

### Demographic Profile, Clinical Presentation, and Tumor Characteristics of HCC Patients of Viral Etiology and Comparison Between HBV and HCV Related HCC

A total of 158 viral-HCC patients suitable for LRT were evaluated. Of these, 68 were excluded (10 refused treatment, 13 refused to participate, 14 lost to follow up, and 31 did not report at 1-month post-LRT) and thus 90 patients were studied. Demographic, clinical profile, and laboratory details of all viral-HCC patients and comparison between HBV-HCC and HCV-HCC are provided in **Table 2**. Viral-HCC patients had a mean age of 52.5 ± 11.4 years and were predominantly males 73/90 (81%). The underlying etiology was HBV in 64/90 (71.1%), HCV 23/90 (25.6%), and 3/90 (3.3%) had dual infection of HBV and HCV. These patients were subjected to various types of LRTs such as RFA, 11/90 (12.2%); PAI, 4/90 (4.4%); and TACE 75/90 (83.3%). Demographic and clinical features comparison of HBV-HCC and HCV-HCC patients showed no significant differences.

**Table 2:** Demographic, biochemical, and clinical profile of study population and comparison between HBV and HCV etiology.

S. No.	Parameters	Viral-HCC (n = 90)	HBV-HCC (n = 64)	HCV-HCC (n = 23)		*p-value
**1**	Age, years	52.5 ± 11.4	51.88 ± 12.24	56.14 ± 8.46		0.14
**2**	Male (%)	73 (81.1%)	56 (87.5%)	14 (60.8%)		**0.006**
**3**	AST, IU/L	61.2 ± 29.3	60.7 ± 29.1	65.8 ± 30.6		0.48
**4**	ALT, IU/L	49.1 ± 29.2	46.9 ± 22.7	48.8 ± 17.5		0.69
**5**	SAP, IU/L	294.1 ± 155.9	305.22 ± 169.7	260.0 ± 107.2		0.24
**6**	S. Bilirubin, mg/dl	1.03 ± 0.63	0.94 ± 0.54	1.3 ± 0.81		**0.02**
**7**	Protein	7.3 ± 0.72	7.26 ± 0.74	7.47± 0.66		0.25
**8**	Albumin, g/dl	3.8 ± 0.63	3.81 ± 0.67	3.83 ± 0.48		0.93
**9**	PT, s	14.3 ± 2.9	14.4 ± 2.78	14.3 ± 3.28		0.88
**10**	Hb, g/dl,	12.1 ± 1.9	12.2 ± 2.1	11.67 ± 1.42		0.24
**11**	TLC (per mm^3^)	5,685.1 ± 2,123.1	5,756.7 ± 2,117	5,532.2 ± 2,284.9		0.67
**12**	PLT × 10^3/^mm^3^	146.4 ± 103.8	159.8 ± 112.8	119 ± 73.6		0.11
**13**	Blood Urea	24.4 ± 8.5	23.8 ± 7.9	26. 5 ± 10.3		0.19
**14**	S. Creatinine, mg/dl	0.83 ± 0.24	0.83 ± 0.23	0.83 ± 0.25		0.89
**15**	AFP, ng/mlMedian, IQR	31.3 (5–254)	13.7 (3.7–253.6)	73.1 (11.6–477.8)		0.59
**16**	Tumor Size, cm	3.71 ± 2.72	3.76 ± 2.95	3.82 ± 2.12		0.93
**17**	Number of TumorSolitary Multiple	56 (62.9%)33 (37.1%)	36 (56.3%) 27 (42.2%)	18 (78.3%) 5 (21.7%)		0.07
**18**	BCLC A BCLC B	48 (53.3%)42 (46.7%)	33 (51.6%) 31 (48.4%)	14 (60.9%) 9 (39.1%)		0.44
**19**	CTP A CTP B	71 (78.9%)19 (21.1%)	49 (76.6%) 15 (23.4%)	19 (82.6%) 4 (17.4%)		0.55
**20**	PST 0PST 1	81 (90%)9 (10%)	55 (85.9%) 9 (14.1%)	23 (100%) 0 (0%)		0.06
**21**	Locoregional TherapyRFAPAITACE	11 (12.2%) 4 (4.4%) 75 (83.3%)	7 (10.9%)2 (3.12%)55 (85.9%)	3 (13.0%) 2 (8.7%) 18 (78.3%)		0.53
**22**	Tumor ResponseComplete ResponsePartial ResponseProgressive Disease	36 (40.0%) 28 (31.1%) 26 (28.9%)	24 (37.5%)20 (31.3%)20 (31.3%)	9 (39.1%) 8 (34.8%) 6 (26.1%)		0.23

All values are expressed as n (%) or (mean ± SD) unless otherwise specified. P-value obtained from t- test and Pearson Chi square shows comparison between HBV-HCC and HCV-HCC.

AST, Aspartate aminotransferase; ALT, Alanine aminotransferase; SAP, Serum alkaline phosphatase; PT, Prothrombin time; Hb, Hemoglobin; TLC, Total leukocyte count; PLT, Platelet count; AFP, Alpha-feto protein; HBV, Hepatitis B virus; HCV, Hepatitis C virus; BCLC, Barcelona Clinic Liver Cancer; CTP, Child-Turcotte-Pugh; PST, Performance Status; RFA, Radio frequency ablation; PAI, Percutaneous acetic acid injection; TACE, Transarterial chemoembolization.

Bold values are statistically significant.

### Tumor Response Assessment at 1 Month Post LRT by MPCT/MRI

**Figure 1:**
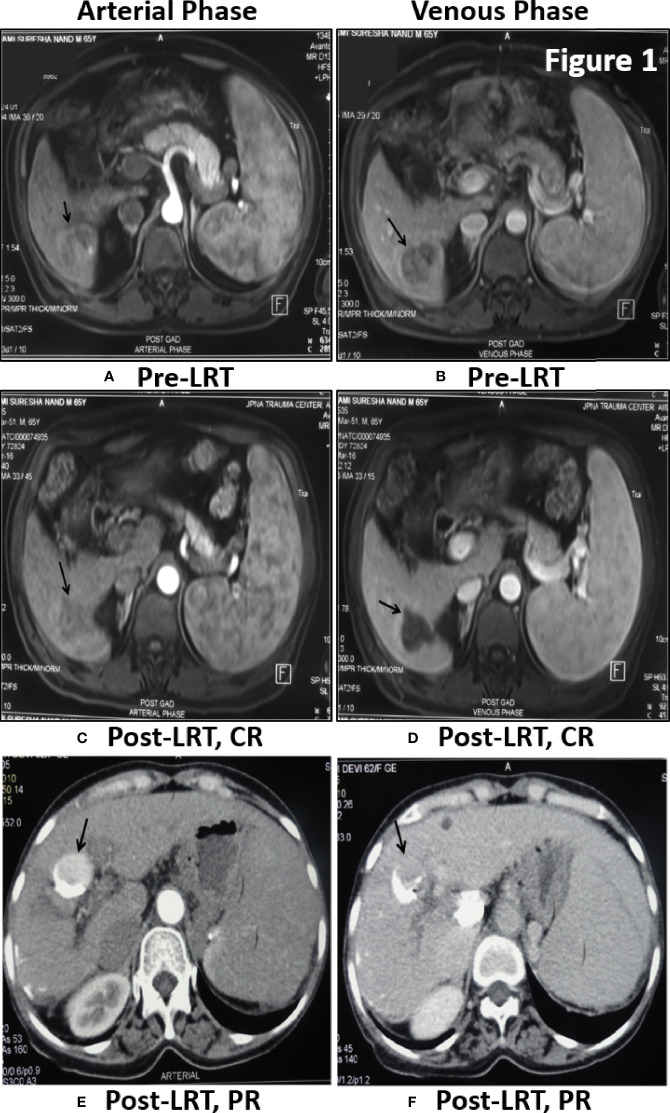
Tumor response assessment by MPCT/MRI of Liver: Post-TACE MRI Scan of 65 yr old male patient of HBV- HCC have shown large solitary HCC in segment 6 showing enhancement in arterial phase **(A)** with washout in venous phase **(B)** before treatment. One month Post-LRT in MRI have shown lack of enhancement in treated tumor in arterial phase **(C)** and venous phase **(D)** is suggestive of Complete Response (CR). Post-TACE CT scan of 62 yr old female patient of HCV-HCC have shown residual enhancing tumor (black arrow) in arterial phase **(E)** with washout in venous phase **(F)**. Dense lipidol seen in the lateral aspect of tumor is suggestive of partial response to LRT.

Tumor response of viral-HCC patients was assessed by MPCT/MRI of liver at 1 month post-LRT. Arterial enhancement (**Figures 1A, C, E** **)** was monitored following washout at venous phase (**Figures 1B, D, F** **)** at both pre- (**Figures 1A, B** **)** and post- (**Figures 1C–F**) LRT. The CR is disappearance of any intra-tumoral arterial enhancement in the target lesions as shown in liver segment 6 of post TACE MRI Scan of HBV- HCC patient (**Figures 1C, D** **)**. The PR is 30% decrease in the sum diameters of viable target lesions as shown in post-TACE CT scan of HCV- HCC patients. Residual enhancing tumor (black arrow) in arterial phase was observed with washout in venous phase (**Figures 1E, F** **)**. The PD is an increase of at least 20% in sum of diameters of viable target lesions whereas, stable disease (SD) is that did not qualify for either PR or PD. In all of our viral-HCC patients, observed tumor response was CR in 40% (36/90); PR in 31% (28/90); PD in 29% (26/90) cases and no SD due to single point response evaluation.

### Relative Expression of oncomiR (miR-21, -221, and -16) at Baseline and Comparison Between Pre- and Post-treatment of Viral-HCC

The miRs-199, -124, and -500 exhibited very low (higher Ct values) or undetectable expression in circulation whereas, miR-21, -221, and -16 were efficiently detected in circulation of viral-HCC patients. Comparison of oncomiR due to viral etiology has shown relatively higher expression in HBV-HCC compared to HCV-HCC, but not found statistically significant (P > 0.05, **Table 3A**). Comparison of oncomiR relative expression in viral-HCC patients at pre- *vs.* post-therapy was also not found significant (P > 0.05, **Table 3B**).

**Table 3A:** Comparison of oncomiRs expression in HBV-HCC and HCV-HCC before LRT.

A. Comparison between HBV-HCC and HCV- HCC			
miRNA	HBV-HCC, Pre therapy (2^-ΔCt^)	HCV-HCC, Pre therapy (2^-ΔCt^)	P value
miR-21	286.9 (22.3–1689)	106.8 (11.4–388)	0.15
miR-221	58.9 (10.9–219.7)	17.5 (6.2–112.9)	0.11
miR-16	680.1 (20.6–5689)	445.6 (93.7–2628)	0.79

**Table 3B:** Comparison of oncomiRs expression before and after LRT in all viral HCC patients.

Comparison of pre- and post-locoregional therapy in viral-HCC			
miRNA	Pre Therapy (2^-ΔCt^)	Post Therapy (2^-ΔCt^)	P value
**miR-21**	219 (15.01–1458)	269.3 (19.8–1446)	0.10
**miR-221**	43.1 (8.1–153.2)	45.1 (10.7–148.4)	0.11
**miR-16**	508.3 (30.0–3901)	266.8 (23.6–9605)	0.27

### Comparison of Clinical Profile According to Different Categories of Response

Comparison of demographic, clinical, and biochemical profile according to tumor response was shown in **Table 4**. As compared to the patients with CR+PR, patients developing PD had slightly higher AFP values at presentation (P = 0.03) in Group 1. The tumor size was significantly larger in patients of PR+PD as compared to CR patients (P = 0.007) in Group 2. Other clinical and biochemical parameters did not show any significant difference among these groups.

**Table 4:** Comparison of demographic, clinical, and biochemical profile among categories of CR, PR, PD, Group 1, and Group 2.

S. No.	Parameters	CR (n = 36)	PR (n = 28)	PD (n = 26)	ANOVA p-value	Group 1 p-value	Group 2 p-value
**1**	Age, years	52.5 ± 11.4	53.3 ± 13.5	51.8 ± 9.7	0.89	0.68*	0.99*
**2**	Male	26 (72.2%)	23 (82.1%)	24 (92.3%)	0.14**	0.08**	0.08**
**3**	AST, IU/L	62.6 ± 30.3	59.8 ± 28.4	60.6 ± 29.9	0.93	0.91*	0.71*
**4**	ALT, IU/L	55.9 ± 39.7	44.4 ± 18.9	44.9 ± 18.2	0.20	0.38*	0.07*
**5**	SAP, IU/L	319.6 ± 184.2	263.3 ± 133.6	292.0 ± 132.4	0.36	0.94*	0.21*
**6**	S. Bilirubin, mg/dl	1.0 ± 0.7	0.98 ± 0.5	1.1 ± 0.6	0.78	0.37*	0.95*
**7**	Protein	7.4 ± 0.6	7.2 ± 0.8	7.3 ± 0.8	0.33	0.87*	0.18*
**8**	Albumin, g/dl	3.8 ± 0.6	3.9 ± 0.5	3.6 ± 0.7	0.21	0.08*	0.44*
**9**	PT, s	13.7 ± 2.4	14.4 ± 2.8	15.2 ± 3.5	0.19	0.10*	0.12*
**10**	Hb, g/dl,	12.1 ± 1.8	11.9 ± 2.1	12.3 ± 2.0	0.84	0.61*	0.98*
**11**	TLC (per mm^3^)	5,784.3 ± 2,223.7	5,648.9 ± 2,264.6	5,582.6 ± 1,870.0	0.93	0.78*	0.72*
**12**	PLT × 10^3^/mm^3^	152.1 ± 122.6	145.1 ± 95.1	139.1 ± 84.6	0.89	0.69*	0.67*
**13**	Blood Urea	24.4 ± 9.0	24.7 ± 9.3	23.9 ± 7.2	0.95	0.77*	0.98*
**14**	S. Creatinine, mg/dl	0.81 ± 0.3	0.84 ± 0.3	0.84 ± 0.2	0.79	0.78*	0.49*
**15**	AFP, ng/mlmedian, IQR	16.3 (6.1–182.4)	9.7 (3.3–92.4)	111 (11.7–1,142)	0.06***	0.03***	0.71***
**16**	Tumor Size, cm	2.8 ± 1.9	4.3 ± 3.1	4.4 ± 2.9	**0.03**	0.14*	**0.007**
**17**	Mass NumberSolitaryMultiple	25 (69.4%) 11 (30.5%)	17 (60.7%) 11 (39.3%)	14 (56.0%) 11 (44.0%)	0.59**	0.67**	0.52**
**18**	HBVHCV	24 (72.7%) 9 (27.3%)	20 (71.4%) 8 (28.6%)	20 (76.9%) 6 (23.1%)	0.89**	0.64**	0.89**
**19**	BCLC ABCLC B	21 (58.3%) 15 (41.7%)	16 (57.1%) 12 (42.9%)	11 (42.3%) 15 (57.7%)	0.25**	0.08**	0.67**
**20**	CTP ACTP B	28 (77.8%) 8 (22.2%)	24 (85.7%) 4 (14.3%)	19 (73.1%) 7 (26.9%)	0.35**	0.49**	0.66**
**21**	PST 0PST 1	32 (88.9%) 4 (11.1%)	26 (92.9/%) 2 (7.1%)	23 (88.5%) 3 (11.5%)	0.83**	0.76**	0.77**

P-value obtained from ANOVA, *t- test, **Pearson Chi square, ***Wilcoxon rank sum test.

Group 1 is comparison of CR+PR from PD patients and Group 2 is comparison of CR from PR+PD patients.

Bold values are statistically significant.

### Trends of Change in miR-21, -221, and -16 Expression According to the Categories of Tumor Response

**Figure 2:**
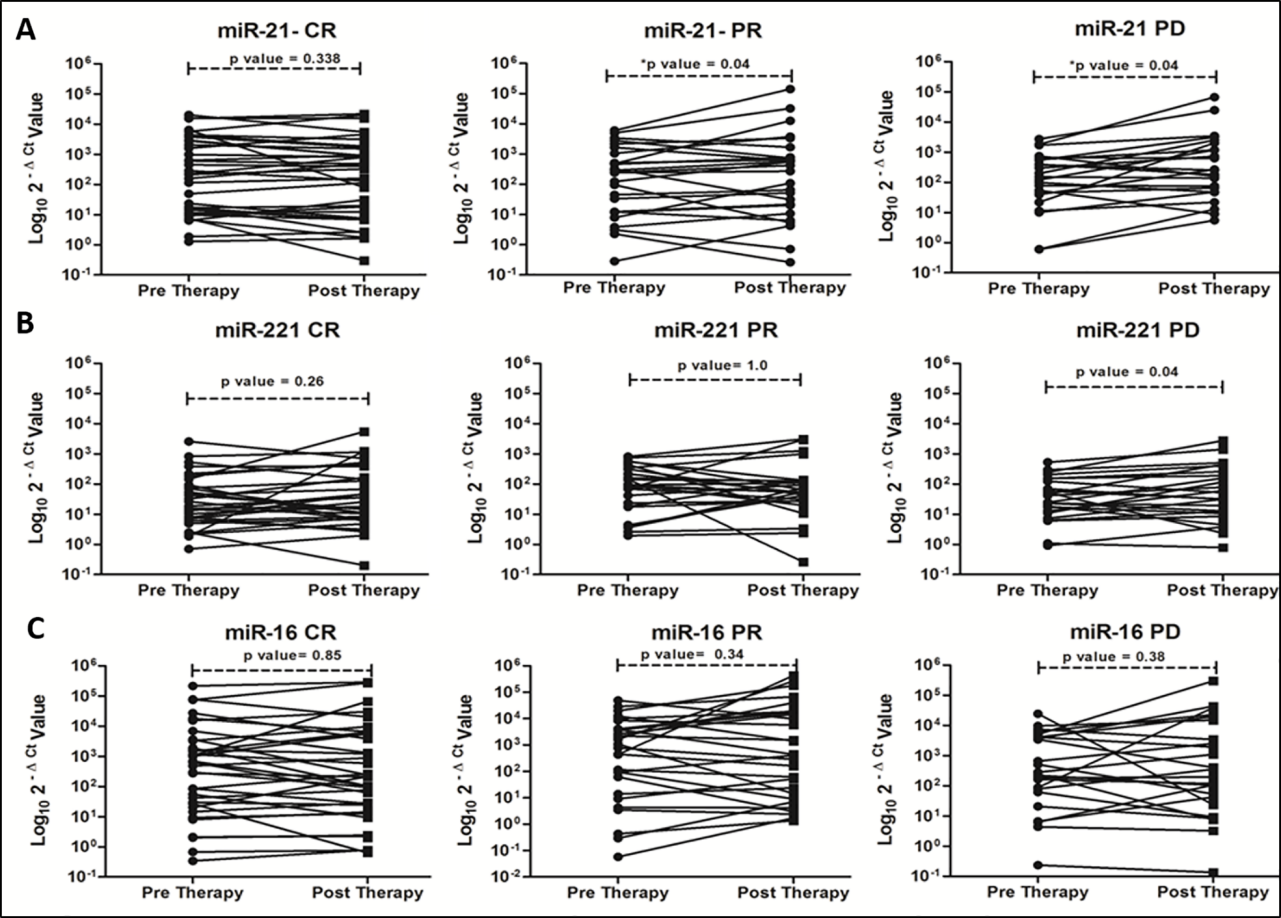
Trends of oncomiR expression in different categories (CR, PR and PD) of tumor response at 1 month post-locoregional therapy in viral-HCC patients for miR-21 **(A)**, miR-221 **(B)** and miR-16 **(C)**.

On correlating with different categories of tumor response, certain trends in relative miRNA expression were noted (**Figure 2**). Significant increasing trend for miR-21 in both PR and PD group (**Figure 2A**, P = 0.045 and 0.040), and for miR-221 in PD group (**Figure 2B**, P = 0.047) were observed. No significant change in miR-16 expression was observed post-LRTs for all three groups (**Figure 2C**).

### OncomiR Fold Changes and AFP Serial Changes in Different Categories of Response at 1 Month Post-therapy

Of all three oncomiRs (miR-21, -221, and -16), post-LRT fold changes of miR-21 alone was found significant. Significant decrease in fold changes of miR-21 occurred in CR patients whereas an increasing trend occurred in PR and PD patients (p = 0.008, **Table 5A**).

**Table 5A:** MicroRNA fold changes in different categories of response in viral-HCC patients following locoregional therapy.

A.	Complete Response	Partial Response	Progressive Disease	
**miRNA**	**FC (median, IQR)**	**FC (median, IQR)**	**FC (median, IQR)**	**P value**
**miR-21**	0.829 (0.40–1.49)	1.62 (0.47–3.85)	2.07 (0.91–9.13)	**0.008**
**miR-221**	1.41 (0.33–2.79)	1.18 (0.24–3.12)	1.71 (0.57–4.28)	0.55
**miR-16**	1.13 (0.26–2.29)	1.77 (0.37–5.66)	0.91 (0.41–4.71)	0.37

Bold values are statistically significant.

Also the levels of median AFP were evaluated in AFP secreting viral-HCC in CR, PR, and PD patients at pre and post LRT (**Table 5B**). Increasing trends of AFP level in PR and PD groups as compared to CR group found significant (p = 0.005) at post-LRT. Whereas significant reduction of AFP levels in patients with complete response (P = 0.007) was observed at post-LRT compared to pre-LRT levels. A slight decrease of AFP levels was seen in PR group and enhanced levels were reported in PD patients. These changes were not significant.

**Table 5B:** Median AFP changes in different categories of response in viral-HCC patients at pre and post LRT.

B. Comparison of AFP pre- and post-locoregional therapy in viral-HCC				
AFP	Complete Response	Partial Response	Progressive Disease	P value
**Pre-LRT**	182.4 (54.2–1,892)	173.6 (78.5–2,149)	300.8 (102.5–2,172)	0.73
**Post-LRT**	32.4 (15.7–78)	123 (30.5–546)	479 (91.4–21,096)	**0.005**
**P-value**	**0.007**	0.28	0.42	

P values calculated by Kruskal Wallis Test (non-parametric).

Bold values are statistically significant.

### Differentiating Group-1 and Group-2 Patients by Post LRT Fold Change of miRNA-21 and -221 Expressions

**Figure 3:**
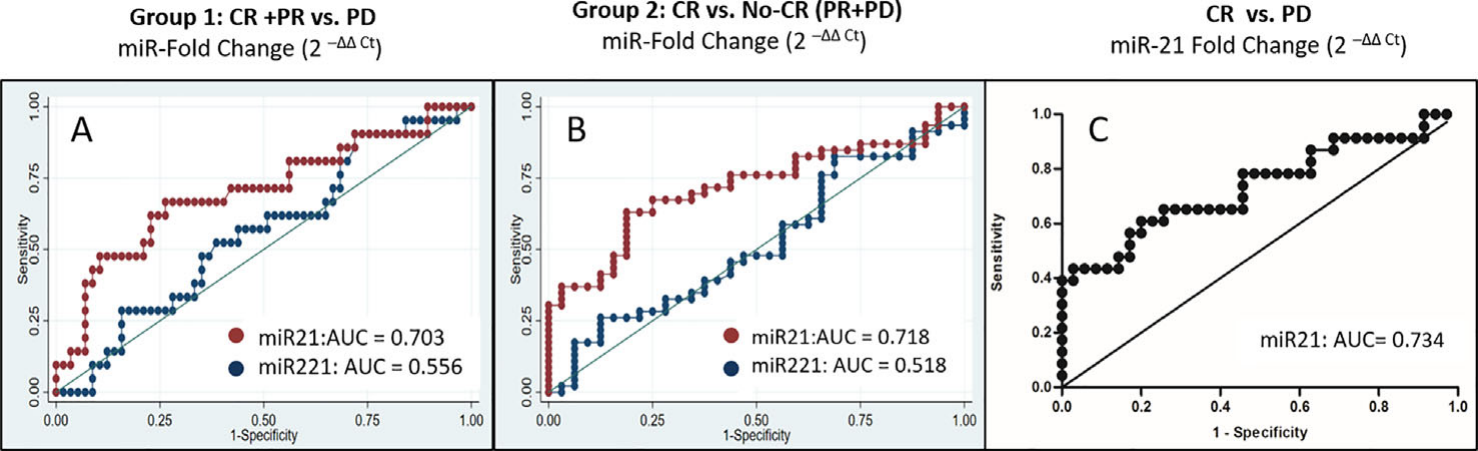
Relative fold changes of miR-21 and -221 in differentiating response to locoregional therapy in viral-HCC patients of **(A)** Group1 (CR + PR from PD), **(B)** Group 2 (CR from PR + PD), and **(C)** CR vs. PD.

Post-LRT fold changes (2^-ΔΔCt^) of miR-21 (red line) and -221 (blue line) expression were analyzed further for predicting response using area under receiver operating characteristic curve (AUROC) analysis for group 1 and 2 patients of viral-HCC (**Figure 3**). To differentiate in group 1 (CR+PR from PD), the miR-21 AUROC was 0.703 (95% CI, 0.540–0.815) at the cut off value of 1.9 fold change (**Figure 3A**). The sensitivity and specificity was 60.9% (38.5–80.3) and 73.8% (60.9–84.2) respectively with positive predictive value 46.7% (28.3–65.7) and negative predictive value 83.3% (70.7–92.1). The miR-221 AUROC was 0.556 (95%CI, 0.441–0.707) and for miR-16, it was 0.512 (95% CI, 0.369–0.655). This indicated that both miR-221 and -16 fold change could not predict response in this study.

The HCC patients with residual disease or not achieving CR are planned for retreatment or repeat LRT. Therefore, viral-HCC patients were further grouped to differentiate CR from PR+PD in group 2 (**Figure 3B**). To differentiate in group 2 (CR from PR+PD), the miR-21 AUROC was 0.718 (95% CI, 0.572–0.799) at the cut off value of 1.4 fold change (**Figure 3B**). The sensitivity and specificity of 63.3% (CI, 48.3–76.6) and 68.6% (CI, 50.7–83.1) respectively with positive predictive value 73.8% (CI, 58–86.1) and negative predictive value was 57.1% (CI, 41.0–72.3). This indicates that if fold change increased beyond 1.4, the response to LRT will worsen and more patients will go towards retreatment. The AUROC increased to 0.734 (95% CI, 0.595–0.873) with 1.33 as a cut off value when compared between CR *vs.* PD patients (**Figure 3C**). The value of AUROC was less than 0.6 for both miR-221 and miR-16 which clearly indicated that both miR-221 and miR-16 could not distinguish CR from the rest of the group.

### Comparison of Serum AFP Ratio and miR-21 Fold Changes Alone or In Combination for Prediction of Response in AFP Producing Viral-HCC Patients

**Figure 4:**
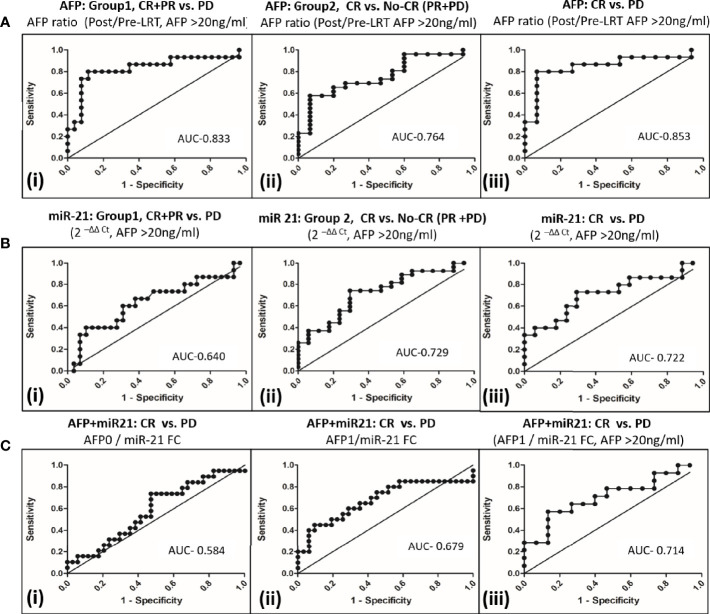
Comparison of AFP and miR-21 fold changes alone or in combination to differentiate tumor response to locoregional therapy in viral-HCC patients. **(A)** AFP ratio (post/pre-LRT) for differentiating viral HCC in AFP secreting patients (AFP>20ng/ml) in group1 (i), CR+PR vs. PD; group 2 (ii), CR vs. No CR(PR+PD) and (iii) CR vs. PD. **(B)** Fold change of miR-21 for differentiating viral HCC in AFP secreting patients in group1(i), CR+PR vs. PD; group 2 (ii), CR vs. No CR(PR+PD) and (iii) CR vs. PD. **(C)** miR-21 and AFP Combination for differentiating viral HCC. Differentiation of CR vs. PD by AFP0/miR-21 FC (i), AFP1/miR-21 FC; (ii) in all HCC patients and AFP1/miR-21 FC in AFP secreting patients (iii).

Prediction of tumor response to differentiate CR+PR from PD, group 1 (**Figure 4Ai**) using serum AFP ratio alone in ROC curve of AFP producer group (n = 45) had shown AUC 0.833 (95% CI, 0.69–0.98) at a cut off 1.23 with 80.0% (95% CI, 51.9–95.7) sensitivity and 88.5% (95% CI, 69.9–97.6) specificity. Serum AFP ratio also discriminated CR from PR+PD, group 2 (**Figure 4Aii**) with AUC of 0.764 (95% CI, 62.0–91.0). The potential of AFP ratio alone to distinguish CR from PD in in AFP producing patients was shown to be highest with an AUROC of 0.853; 95% CI, 0.70–1.0 (**Figure 4Aiii**). The cut off value was 1.16 with 80.0% (95% CI, 51.9–95.7) sensitivity and 93.3% (95% CI, 68.1–99.8) specificity. This indicated that increased AFP ratio ≥1.2 likely progress to PD at post-LRT.

The miR-21 fold changes in AFP producing HCC patients can also predict post-LRT tumor response with AUC of 0.640 (95% CI, 0.459–0.822) for CR+PR *vs.* PD, group 1 (**Figure 4Bi**); with AUC, 0.729 (95% CI, 0.579–0.881) for CR *vs.* PR+PD, group 2 (**Figure 4Bii**) and AUC, 0.722 (95%, 0.539–0.904) for CR *vs.* PD patients (**Figure 4Biii**). Combination of AFP with miR21FC at pre-LRT (AFP0/miR21FC) and 1 month post-LRT (AFP1/miR21FC) was evaluated (**Figure 4C**). AFP at 1 month post-LRT in combination with miR-21FC had shown significant AUROC differentiating CR *vs.* PD for all HCC patient (AUC, 0.679; 95% CI, 0.515–0.843, **Figure 4Cii**) and only AFP secreting HCC patient (AUC, 0.714; 95% CI, 0.523–0.906, **Figure 4Cii**). Differentiation CR *vs.* PD using miR-21FC in combination with AFP at base line (AFP0) was not found significant with AUC below 0.60 (**Figure 4Ci**). There was no added advantage differentiating CR *vs.* PD by AFP ratio and miR-21 FC. But both miR-21 and AFP ratio were equally capable of discriminating CR from other tumor responses and can be used as an independent predictor.

### Survival Analysis

**Figure 5:**
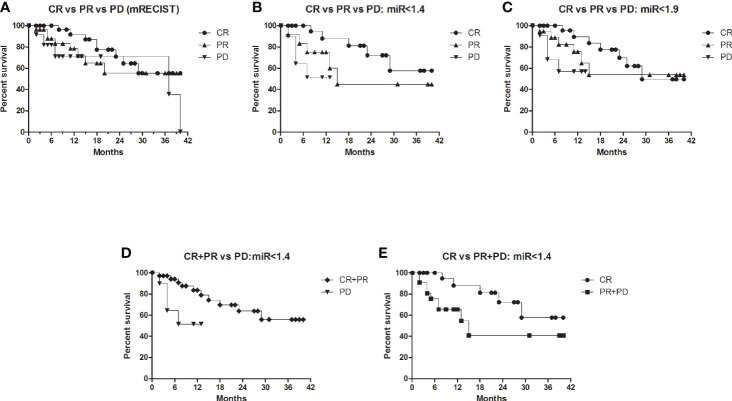
Survival curve comparison of CR, PR, and PD patients at 42-months post-LRT with miR-21 fold change. Comparison among CR vs. PR vs. PD as per mRECIST criteria in all HCC patients **(A)**, HCC patients with miR-21 FC cut off value < 1.4 **(B)**, HCC patients with miR-21 FC cut off value < 1.9 **(C)**, HCC patients with miR-21 FC cut off value < 1.4 in group 1, CR + PR vs. PD **(D)**, HCC patients with miR-21 FC cut off value < 1.4 in Group 2, CR vs.PR + PD **(E)**.

The differences in survival among different groups were calculated using log-rank test (Mantel-Cox test) by plotting Kaplan-Meier survival curve. Initial tumor response (CR *vs.* PR *vs.* PD) at 1 month that drive the survival outcome at 42-month post-LRT were mentioned for all patients (**Figure 5A**) and at cut off fold change of miR <1.4 (53.3%, n = 48, **Figure 5B**) and miR <1.9 (66.6%, n = 60, **Figure 5C**).

The patients with miR-21 fold change cut off <1.4 had shown significant (p = 0.049) survival differentiating CR, PR, and PD (**Figure 5B**). Poor survival was observed in PD patients when compared with CR+PR (P = 0.023) in group 1 (**Figure 5D**, miR <1.4 fold change) whereas, significant better survival was observed in CR patients when compared with PR+PD patients (P = 0.04) in group 2 (**Figure 5E**, miR <1.4 fold change).

### Luciferase Reporter 3’-UTR Assay for Determination of microRNA Target Specificity Modulating mTOR Pathway

**Figure 6:**
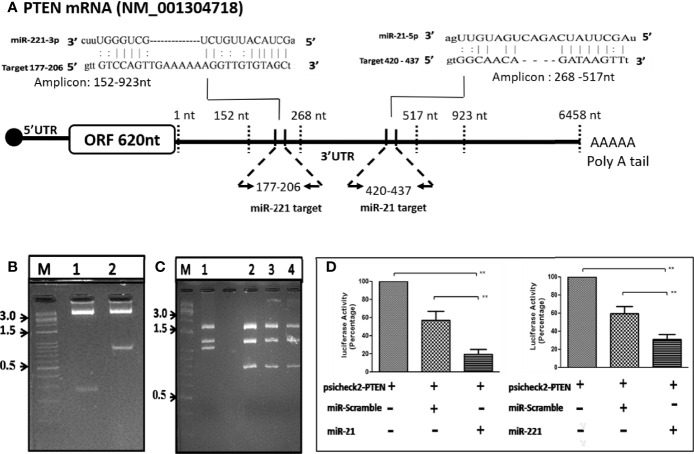
OncomiR target validation by Luciferase reporter 3’-UTR assay. **(A)** Schematic depiction of PTEN UTR as miR-21 and -221 targets through bioinformatic analysis. **(B)** Direction cloning of PTEN 3’-UTR in psiCheck-2 vector as target for miR-21 (Lane 1) and miR-221 (Lane 2) and confirmed by double digestion with XhoI/NotI. **(C)** Cloning of short hairpin miR precursor in psiRNA-h7SKGFPzeo vector at BbsI. Positive clones are confirmed by digestion with SpeI (Lanes 1, no insert; 2, miR 21; 3, miR-221; 4, miR-Scr). **(D)** Luciferase reporter 3’-UTR assay for target validation of miR-21 and miR-221. **P ≤ 0.01.

The putative target binding sites of miR-21 and -221 were bioinformatically predicted to UTR of PTEN gene (**Figure 6A**), a negative modulator of mTOR pathway. The UTR region flanking 152-923nt of PTEN was cloned downstream to luciferase ORF in psiCheck-2 reporter vector (**Figure 6B**) for determination of target specificity. For miRNA production in cell, short hairpin RNA (shRNA) insert containing mature miRNA sequence were cloned in psiRNAh7skGFPzeo vector (**Figure 6C**). This vector contains RNA polymerase III promoter h7sk to transcribe short RNA from cloned insert after transfection which naturally processed for production of mature miRNA. Transfection of psiRNAh7skGFPzeo miR-21, -221 and miR-Scr confirmed miRNA production by real time PCR and transfection psiCheck-2 produced renilla luciferase as determined by luciferase assay. The UTR assay following co-transfection had shown significant reduction of luciferase activity by candidate miR-21 and -221 as compared to miR-Scr and PTEN-UTR alone. Percentage inhibition of luciferase activity by candidate miR-21 and miR-221 were 80.6% (P = 0.002) and 72% (P = 0.004) respectively as compared to PTEN-UTR alone (**Figure 6D**).

### Effect of miR and anti-miR on mTOR Pathway in microRNA Overexpressing Stable Cell Line

**Figure 7:**
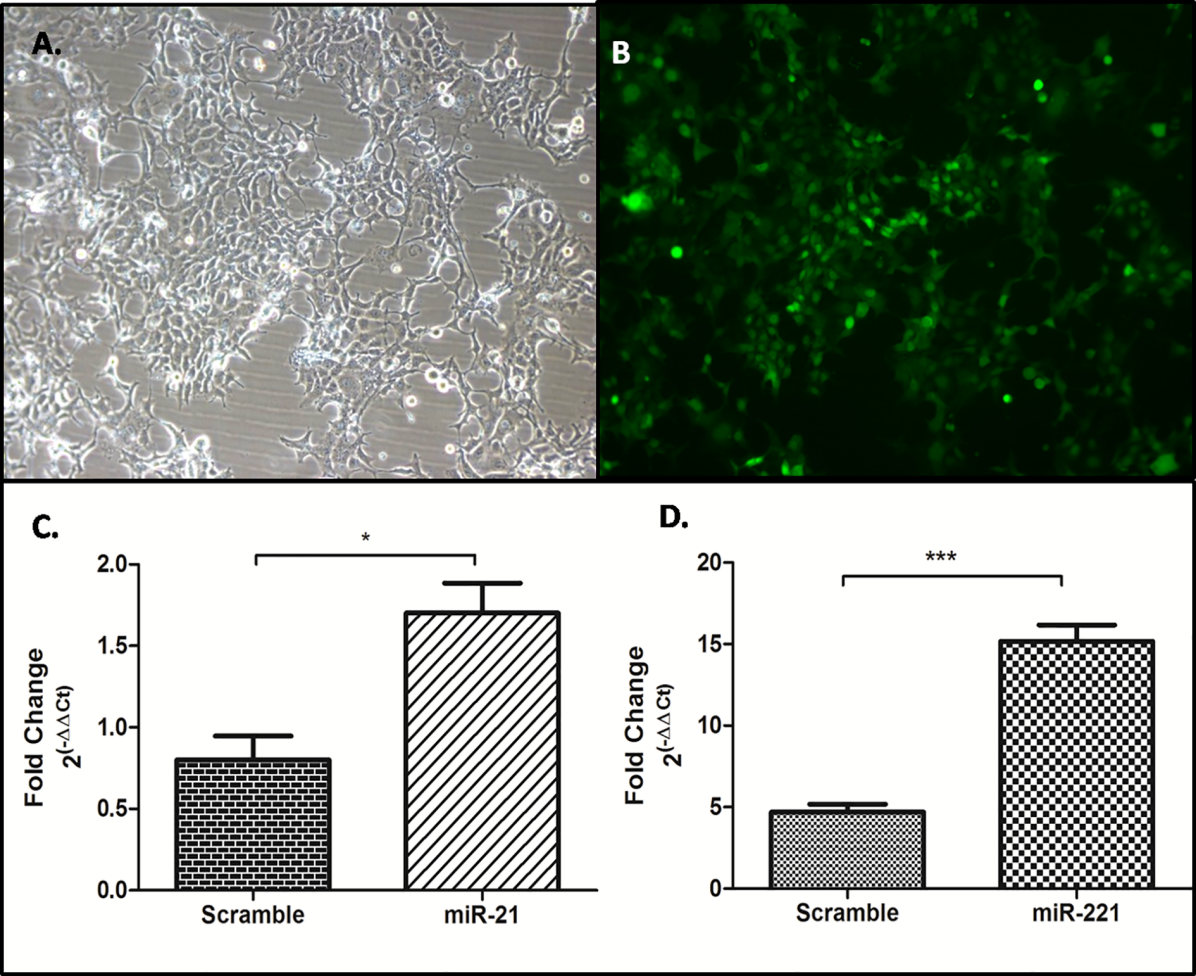
Generation of stable cell line overexpressing miR-21, -221, and miR-Scr. Stable selection of Huh7 cell lines were carried out by zeocin selection **(A)** and confirmed by reporter GFP expression **(B)**, Overexpression of miR-21 **(C)**, and miR- 221 **(D)** compared to miR-Scr was checked by miR specific SYBR green based real time PCR after normalization with U6 snRNA. (*P ≤ 0.05), (***P ≤ 0.0001).

Generation of Stable Huh-7 cell lines expressing miR-21, -221, and miR-Scr was achieved through zeocin selection and confirmation by GFP reporter gene expression (**Figures 7A, B** **)**. Significant increase of miR-21 and -221 expression was observed by Real-time PCR in psiRNA-h7SKGFPzeo-miR-21 (**Figure 7C**) and -miR-221 (**Figure 7D**) stable cell lines as compared to miR-Scr cell line.

**Figure 8:**
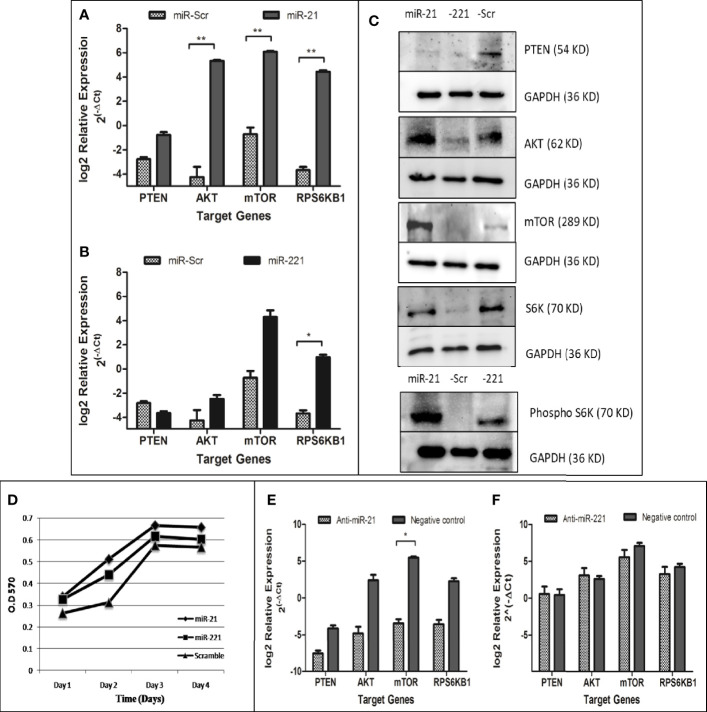
Functional evaluation of PTEN/AKT/mTOR pathway activation in oncomiR overexpressing cell lines. Relative expression of mTOR pathway associated genes (PTEN, AKT, mTOR, and RPS6KB1) in miR-21 **(A)** and miR-221 **(B)** cell line as compared to miR-Scr cell line following normalization with beta-actin. Immunoblot of PTEN, AKT, mTOR, total/phosphorylated p70S6K, and GAPDH in miR-21, -221, and Scr overexpressing cell line **(C)**. Effect of oncomiR on cell proliferation was assessed by MTT assay **(D)**. Effect of antimiR-21 **(E)** and antimir-221 **(F)** on mTOR pathway was compared to negative antimiR control for relative gene expression of PTEN, AKT, mTOR, and RPS6KB1 by real-time PCR. (*P ≤ 0.05), (**P ≤ 0.01).

The PTEN/Akt/mTOR signaling pathway is important for cell proliferation, growth, and angiogenesis. The PTEN gene, a key negative regulator mTOR pathway, is main target for several miRNAs. Dysregulation of these miRNA results in activation of mTOR pathway and enhanced cell proliferation. The effect of dysregulated miRNA on mTOR pathways was studied for downstream gene expression by real time PCR (**Figures 8A, B** **)**, by western blot (**Figure 8C**), and for cell proliferation by MTT assay (**Figure 8D**). Relative gene expression of PTEN/AKT/mTOR signaling pathway had shown increased expression of AKT, mTOR, and RPS6KB1 in miR-21 cell line as compared to cell line expressing miR-Scr (**Figure 8A**). In case of miR-221 overexpressing cell line, only RPS6KB1 levels were found to be raised in comparison to scramble cells (**Figure 8B**). Immunoblot of cell lysate obtained from miR-21, -221 stable cell line had shown significant reduction of PTEN protein and increased phosphorylation of S6K protein (**Figure 8C**) as compared to miR-Scr stable cell line. The level of Akt and mTOR also increased in miR-21 overexpressing cell line. Increased phosphorylation relative to total S6K protein is an indicator of mTOR signaling pathway activation, which occurred in both miR-21 and miR-221 expressing stable cell lines (**Figure 8C**). Increased cell proliferation by MTT assay was observed in both miR-21 and miR-221 stable Huh-7 cells as compared to miR-Scr (**Figure 8D**). AntimiR specific to miR-21 resulted in reduction of PTEN, AKT, mTOR and RPS6KB1 gene relative to antimiR negative control and the reduction of mTOR gene expression was found significant (P = 0.02) (**Figure 8E**). AntimiR specific to miR-221 did not show any significant change to the downstream gene expression (**Figure 8F**).

## Discussion

Loco-regional therapies (LRTs) including chemoembolization, radioembolization, and acetic acid injections remain mainstay of HCC treatment. Dysregulated oncomiRs which correlated well with imaging modalities of tumor response can be used as biomarker for monitoring therapeutic response. Previous studies have shown correlation of high miR-335 levels with higher response rate at 30 days post-TACE in HCC patients (23). Significant association of miR-200a expression with prognosis was observed in HCC patients subjected to TACE (24). This shows prognostic potential of oncomiR during therapy. We have chosen six oncomiRs (miR-21, 221,16,199,124, and 500) known to be dysregulated in HCC. These are considered oncomiRs because they (miR-124 and -199) are downregulated in HCC and target primarily oncogenes including PIK3CA (25), AKT (26), and PAK4 (27).The upregulated oncomiRs target tumor suppressor genes such as PTEN (28), PDCD4 (29), TPM1 (30), and MAP2K3 (31) for miR-21; p27KIP1 (32), FOXO3A (33), and PTEN (34) for miR-221 and PTEN (35) for miR-500. The main target tumor suppressor PTEN gene is a potent negative regulator of mTOR pathway usually activated in HCC and enhances cell proliferation and carcinogenesis. Oncogenic role of miR-16 has been reported both for its down-regulation targeting oncogenes such as BCL2, CCND1 (36), and up-regulation targeting tumor suppressor gene (37).

During screening of these miRNA at baseline and post therapy, we had observed that miR-124, -199, and miR-500 were not detected efficiently. The upregulated oncomiRs (miR-21 and -221) have shown increasing trends in PR and PD groups (**Figure 2**). Increased miR-21 expression was associated with post-transplant HCC recurrence in HBV-HCC (38, 39), while upregulation of miR-221 contributed to hepatocarcinogenesis (40) in earlier studies (41). Both up (42) and downregulation (43) of miR-16 have been reported in tumor tissue of HCC patient as compared to peri-tumoral tissue. Increased miRNA-16 expression has been reported in HCV associated HCC (44). But any changes in miRNA expression associated with locoregional therapy have not been reported so far. The AUROC curve for miR-21 and -221 fold changes were plotted for prediction of tumor response (**Figures 3A–C**), miR-21 showed better prediction ability differentiating CR+PR from PD (AUC, 0.703 with 60.9% sensitivity and 73.8% specificity at cut-off ≥1.9 fold change of miR-21) at 1 month post-LRT. No-CR (PR+PD) patients require repeat or additional treatment and differentiating ability miR-21 fold changes was found at ≥1.4 fold change cut off with AUC, 0.718, 63.3% sensitivity and 68.6% specificity and ≥1.33 fold change cut off with AUC, 0.734 with 0.652 sensitivity and 0.657 specificity for differentiation of CR from PD. Positive correlation of higher miR-21 expression was observed with cirrhosis and tumor stages in earlier studies (45) and also considered independent predictor for advanced stage HCC with portal vein thrombosis (46).

So far, AFP is the only available marker for HCC diagnosis and recent studies have shown its new role as a prognostic marker as well. AFP ratio (post/pre -therapy) <1 indicated decline and >1 indicated rise of AFP in AFP secreting HCC (>20 ng/ml). Decrease in AFP ratio was observed after treatment and increased AFP ratio associated with HCC progression (47). In our study, AUROC of AFP ratio could also predict tumor response differentiating CR+PR from PD (AUC, 0.833; 95% CI, 0.69–0.98, **Figure 4Ai**), CR from PR+PD (AUC, 0.764; 95% CI, 62.0–91.0, **Figure 4Aii**), and CR from PD (AUC, 0.853; 95% CI, 0.70–1.0, **Figure 4Aiii**). The AFP ratio cut off was 1.23 with sensitivity, 80.0% and specificity, 88.5%. Earlier studies have shown direct association of higher AFP levels (>200 ng/ml) with tumor size, stage, and HBsAg positivity in viral-HCC (48). More marked and detectable serial changes of AFP following treatment can also be considered predictive markers. Increased levels of miR-21 had shown poor prognosis in earlier studies. Short span of survival was associated with HCC post operation in patients with higher miR-21 levels (49) whereas better overall survival was associated with HCC patients with lower miR-21 expression (50). In another study, higher expression of serum exosomal miRNA-21 was correlated with low survival rate of HCC (51). In our study the CR patients have shown better survival with miR-21FC value less than 1.4 cut off following 42 months post LRT (**Figure 5**).

Target of miR-21 and miR-221 predicted in UTR of PTEN and confirmed by luciferase-UTR reporter assay (**Figure 6**). PTEN targeting by microRNAs result in mTOR pathway activation and dysregulation of these microRNAs lead to an uncontrolled cell proliferation leading to cancer. Functional role of upregulated microRNA was studied by generating stable cell lines overexpressing miR-21, -221, and miR-Scr (**Figure 7**). Increased proliferations of Huh7 cell were observed by MTT assay (**Figure 8D**). Comparison of miR-21 and miR-221 cell line has indicated prominent activation of PTEN/AKT/mTOR pathway by miR-21 as evidenced from reduced protein expression of PTEN, significant increased expression of AKT, mTOR, and phosphorylated p70S6K with marked cell proliferation than miR-221 stable cell line (**Figure 8C**). Earlier studies in cancer confirmed miR-21 and miR-221 mediated AKT/PTEN/mTOR signaling cascade activation by western blot (34). In our study no change or reduced expression of PTEN mRNA levels (**Figures 8A, B** **)** were noted as compared to miR-Scr indicating overexpression of both miRNAs had less effect on PTEN mRNA degradation. However increased expression of downstream effector gene Akt, mTOR, and RPS6KB1 was observed with higher fold change in miR-21 as compared to miR-221 expressing cells. This indicates that interaction of miR-21 with PTEN is more effective. We have confirmed that overexpression of miR-21 and -221 plays a role in tumorigenesis through activation of mTOR pathway in our study. Transfection of anti-miR-21 specifically reduced expression levels of AKT, mTOR, and RPS6KB1 as compared to control inhibitor in the other group (**Figure 8E**). This result suggests that inhibition of miR-21 activity in hepatoma cells also reduces the PTEN/AKT/mTOR pathway activity.

To conclude, miR-21, a proven oncomiR, is found to be useful predicting therapeutic response to LRT in HCC patients irrespective of their AFP secretion. Diagnostic accuracy of miR-21 is also comparable to AFP in HCC patients and also found to correlate well with overall survival. In HCC, miR-21 overexpression may result in mTOR pathway activation and can be targeted using antimir. Focusing only on mTOR pathway is the limitation of our study, as several other signaling pathways such as RAS/MAPK, WNT/beta catenin, Hedgehog, and Notch signaling pathways are also involved in hepatocarcinogenesis (52). *In situ* expression of miR-21 in liver tissue was not feasible in the recruited patients, which may be considered as limitations of this study.

## Data Availability Statement

The raw data supporting the conclusions of this article will be made available by the authors, without undue reservation.

## Ethics Statement

The studies involving human participants were reviewed and approved by Ethical Committee (IEC), All India Institute of Medical Sciences (IESC/T-121/25.02.2015). The patients/participants provided their written informed consent to participate in this study.

## Author Contributions

NN: Concept, data acquisition, draft writing. SP: Data acquisition, draft revision. SV: Statistical analysis. DY: Data acquisition. SK: Data acquisition. AS: Draft writing. SG: Data acquisition. SKA: Concept, draft writing. S: Study concept, draft writing. BN: Concept, data acquisition, draft writing. All authors contributed to the article and approved the submitted version.

## Funding

The Council of Scientific and industrial Research, Government of India (CSIR/9/6 (0456)/2015 EMR-I), and Indian Council of Medical Research (ICMR/5/13/12/2019-NCD-III) had provided funding supported for Senior Research Fellowship and for this study to carry out research at the Department of Gastroenterology, All India Institute of Medical Sciences, New Delhi.

## Conflict of Interest

The authors declare that the research was conducted in the absence of any commercial or financial relationships that could be construed as a potential conflict of interest.
